# EQD2-adjusted mean lung dose and symptomatic pneumonitis after thoracic radiotherapy following immune checkpoint inhibitors in lung cancer: a single-center retrospective cohort study

**DOI:** 10.3389/fonc.2026.1923622

**Published:** 2026-10-09

**Authors:** Xiqing Li, Libin Liu, Nuo Zheng, Nila Wang, Tingxiong Xiao, Xiaolong Lin, Haidun Chen, Qin Shi, Yuyin Le

**Affiliations:** 1 Department of Radiation Oncology, Fuzhou Pulmonary Hospital of Fujian Province, Teaching Hospital of Fujian Medical University, Fuzhou, Fujian, China; 2 Department of Oncology, Fuzhou Pulmonary Hospital of Fujian Province, Teaching Hospital of Fujian Medical University, Fuzhou, Fujian, China

**Keywords:** immune checkpoint inhibitors, lung cancer, mean lung dose, radiation pneumonitis, thoracic radiotherapy, treatment interval

## Abstract

**Background:**

Evidence on pneumonitis after thoracic radiotherapy following immune checkpoint inhibitor (ICI) exposure remains limited. We examined associations with voxel-wise EQD2 mean lung dose (MLD_EQD2_) and the ICI–radiotherapy interval.

**Methods:**

This single-center retrospective cohort included 98 patients in a 90-day competing-risk analysis of grade ≥2 radiation pneumonitis (CTCAE), comprising 26 pneumonitis events, 70 event-free observations, and two deaths before pneumonitis. Archived DICOM doses were converted voxel-wise using α/β = 3 Gy. Cumulative incidence was estimated using the Aalen–Johansen method, and Fine–Gray regression included continuous MLD_EQD2_ and treatment interval. Supplementary logistic analyses included 96 patients.

**Results:**

The 90-day pneumonitis cumulative incidence was 26.5% (95% CI 17.7%–35.3%). The adjusted subdistribution hazard ratio (SHR) for MLD_EQD2_ was 1.339 per Gy (95% CI 1.113–1.612; P = 0.002). The interval SHR was 0.784 per 10 days (95% CI 0.639–0.963; P = 0.020). Physical MLD showed a consistent association (SHR 1.197 per Gy, 95% CI 1.036–1.382; P = 0.015).

**Conclusion:**

Higher MLD_EQD2_ and shorter treatment intervals were associated with grade ≥2 symptomatic pneumonitis in this treatment sequence. Clinical ascertainment and dose-reconstruction uncertainties limit interpretation. The absence of an ICI-naive comparator precludes conclusions about ICI-specific radiosensitization. Prospective validation is required before these associations can inform dose constraints or treatment scheduling.

## 1 Introduction

Lung cancer remains a leading cause of cancer-related morbidity and mortality worldwide (1). Programmed cell death protein 1/programmed death-ligand 1 (PD-1/PD-L1) blockade has changed the management of locally advanced lung cancer. These agents are increasingly combined with thoracic radiotherapy in clinical practice (2, 3). Thoracic radiotherapy also remains important in other selected lung cancer settings (4, 5). As radiotherapy after ICI exposure becomes more common, pulmonary toxicity is an important consideration in treatment planning (6, 7).

Radiation pneumonitis (RP) is a major dose-limiting toxicity of thoracic radiotherapy. Symptomatic RP may interrupt cancer treatment, require medical intervention, and occasionally progress to life-threatening respiratory compromise (8, 9). Conventional risk assessment relies largely on dose-volume histogram (DVH) parameters, particularly mean lung dose (MLD) and V20, the lung volume receiving ≥20 Gy. Established constraints were derived primarily from cohorts treated before widespread immunotherapy use (10, 11). Retrospective studies have proposed thresholds for patients receiving immunotherapy, but prospective confirmation is needed before these replace institutional constraints (7, 12). Lung dose, treatment sequence, and the interval between treatments may jointly influence pneumonitis risk (13, 14).

Much of the available evidence concerns chemoradiotherapy followed by consolidation immunotherapy or concurrent treatment, whereas thoracic radiotherapy after prior ICI exposure is less well characterized (15–17). Treatment selection and systemic therapy exposure may influence observed associations between dose, timing, and pneumonitis (6, 18, 19). Estimates specific to this sequence could therefore inform interpretation of pulmonary toxicity in an increasingly encountered clinical setting.

The limited number of events in small cohorts constrains multivariable analysis and prediction-model development (20, 21). A parsimonious association model can address the primary question while limiting model complexity. Supplementary internal validation can characterize the performance and uncertainty of exploratory prediction models.

We evaluated grade ≥2 symptomatic pneumonitis after thoracic radiotherapy in patients previously exposed to ICI. The primary objective was to estimate associations with voxel-wise EQD2 lung dose and the continuous ICI–radiotherapy interval, accounting for competing mortality.

## 2 Materials and methods

### 2.1 Study design and cohort

This single-center retrospective cohort study screened 218 consecutive patients who received thoracic radiotherapy between October 2024 and October 2025; all were treated with volumetric modulated arc therapy (VMAT). Exclusions comprised no prior ICI exposure (n = 109), mediastinal lymph-node-only irradiation (n = 4), unavailable evaluable follow-up (n = 6; no clinical or imaging assessment was recorded after radiotherapy completion, precluding evaluation of the 90-day endpoint), and prior thoracic radiotherapy (n = 1). The remaining 98 patients formed the primary cohort (**Figure 1**). Within 90 days, 26 patients developed RP, 70 remained event-free, and two died before RP. All 98 patients were included in the competing-risk analysis. Supplementary logistic analyses included the 26 patients with RP and the 70 patients with complete event-free follow-up (n = 96). Follow-up was censored at database lock on 18 June 2026.

**Figure 1:**
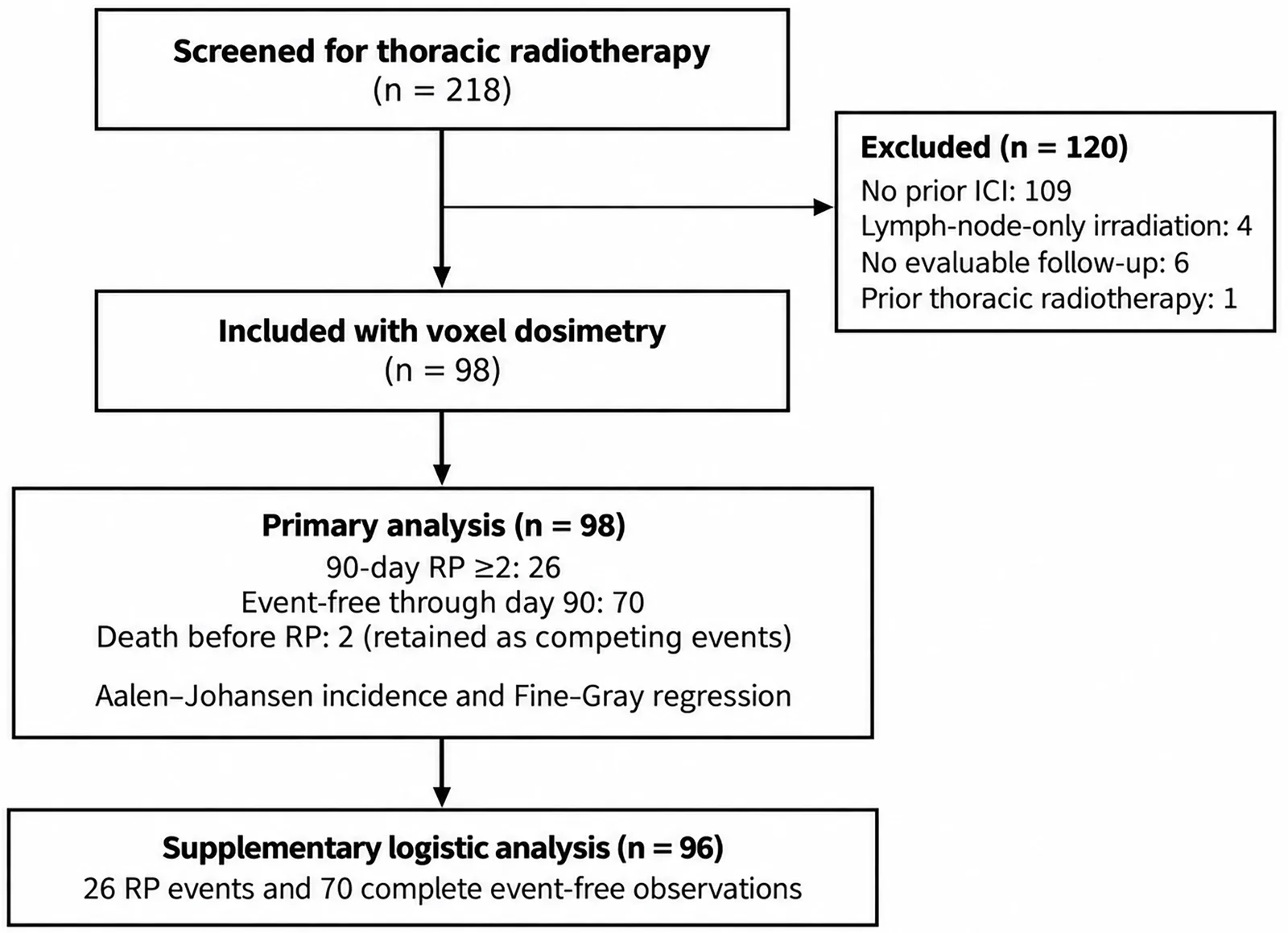
Patient selection and analysis populations. All 98 eligible patients were included in the primary Aalen–Johansen and Fine–Gray analyses. Supplementary logistic analyses included 96 patients with RP or complete 90-day event-free follow-up.

### 2.2 Variables and definitions

Clinical variables included age, sex, smoking status, baseline pulmonary disease, ECOG performance status, histology, stage, ICI class, treatment interval, fractionation, target volumes, and pretreatment hematologic indices. The ICI–radiotherapy interval was measured in calendar days from the last ICI administration to the first radiotherapy fraction; zero indicated same-day treatment. One ICI administration during radiotherapy was documented, but treatment records were insufficient to determine during-radiotherapy ICI exposure consistently across the cohort. Post-radiotherapy ICI exposure was not collected. Specific ICI agents and the period covered by cycle counts could not be reliably determined. Baseline pulmonary disease was recorded as a composite variable, without systematic differentiation of COPD, emphysema, and interstitial lung disease. Histology was categorized as NSCLC, SCLC, mixed carcinoma, or other. The pretreatment hematologic index analyzed was the pre-radiotherapy lymphocyte count (preLYM). Post-radiotherapy lymphocyte decline (LYMdrop) was not included in any model: because the timing of post-radiotherapy blood sampling relative to radiation pneumonitis (RP) onset could not be reliably established from the retrospective records, LYMdrop values may have been measured after RP diagnosis and after initiation of corticosteroid therapy, raising the possibility of reverse causation.

### 2.3 Radiotherapy planning and delivery

Treatment planning was performed in Monaco version 6.0 (Elekta AB, Stockholm, Sweden), and all patients were treated with VMAT. Motion management consisted of 4D-CT in 17 patients and respiratory gating in eight; no specific motion-management technique was documented in the remaining 73 patients. Archived radiotherapy dose, plan, and structure data were used for retrospective dose reconstruction. The lung contour definition was verified for all 98 patients. In every case, the analysis volume comprised bilateral lungs excluding the GTV or ITV, rather than the PTV. The archived combined-lung ROI already incorporated this target exclusion and was used directly, without any further subtraction of target structures. The lung contour was mapped to the native dose grid, and voxel volumes were used for dose-volume calculations. For incomplete courses, the planned dose distribution was scaled according to the number of fractions delivered, assuming an unchanged spatial dose distribution across fractions. Detailed target-margin definitions, nodal inclusion criteria, dose-calculation algorithm and settings, numerical organ-at-risk objectives, and whether low-dose lung volumes such as V5 were explicitly constrained could not be reliably recovered from the retrospective planning records. Technical reconstruction details are provided in the **Supplementary Methods**.

For total physical dose D_i_ at lung voxel i and completed fraction count n, voxel-wise EQD2 was calculated as:


$$EQD2_{i}=\frac{D_{i}\times \;(\frac{D_{i}}{n}+\;3)}{5}$$

assuming α/β = 3 Gy. The volume-weighted mean was:


$$MLD_{EQD2}=\frac{\Sigma_{i}w_{i}EQD2_{i}}{\Sigma_{i}w_{i}}$$

where w_i_ is voxel volume. Vx_EQD2_ was defined as the percentage of the analysis lung volume in voxels with EQD2 ≥ x Gy, where x was 5, 10, 20, or 30 Gy. Physical MLD and Vx were calculated from the same ROI before EQD2 conversion. Because the EQD2 transformation is nonlinear, conversion was performed before averaging; converting the organ-level physical MLD would not yield the same value.

Fraction sizes ranged from 1.5 to 10 Gy, and completed courses comprised 3–30 fractions. Thirteen patients received fractions exceeding 3 Gy. Treatment intent and stereotactic treatment classification could not be reliably determined from the available records.

### 2.4 Endpoint definition and ascertainment

The primary endpoint was clinically recorded grade ≥2 symptomatic radiation pneumonitis (RP) within 90 days after radiotherapy completion (the date of the last delivered fraction). The event date was the date of the diagnostic chest CT on which RP was first documented. Imaging was performed at clinically indicated rather than protocol-specified intervals. Recorded event dates may therefore lag symptom onset. Severity followed the Common Terminology Criteria for Adverse Events (CTCAE) v5.0 (22). Grade 2 was defined by symptoms requiring medical intervention and limiting instrumental activities of daily living. Radiographic changes below this threshold were analyzed as event-free. Recorded grades were used as documented, because the available detail was insufficient for retrospective regrading. Severity and etiologic attribution were considered separately, because radiation-associated, immune-mediated, infectious, and mixed pneumonitis may overlap after ICI exposure. Attribution reflected routine clinical practice, in which predominantly in-field opacities concordant with the isodose distribution supported a radiation-associated cause, whereas diffuse bilateral or out-of-field patterns raised concern for immune-related pneumonitis. Chinese multidisciplinary consensus recommendations provide clinical context for pneumonitis differentiation and management (23). Infection workup and etiologic documentation were incomplete, and independent blinded adjudication was not established. Grades and event dates were abstracted from contemporaneous clinical records using a standardized extraction process; however, the original clinical adjudication itself was not standardized. Because field concordance contributed to attribution, misclassification may be differential with respect to dose, which could inflate or otherwise distort the observed dose association.

### 2.5 Statistical analysis

Follow-up began at radiotherapy completion and ended at the first RP event, death before RP, last valid observation, or day 90. Death before RP was treated as a competing event; deaths occurring after RP did not alter the preceding endpoint. Aalen–Johansen estimates described cumulative incidence in all 98 patients. Fine–Gray regression estimated subdistribution hazard ratios (SHRs) and Wald 95% confidence intervals for MLD_EQD2_ per Gy and treatment interval per 10 days. The two-predictor model was selected after initial data analysis and was not prospectively specified. Covariates were not selected using univariable P values. Model variables were complete, and no imputation was performed. Patient characteristics were summarized descriptively by outcome group.

### 2.6 Model diagnostics and supplementary internal validation

Predictor-by-event-time/90 interactions assessed proportional subdistribution hazards, and a centered quadratic MLD term assessed departure from linearity. An MLD-adjusted restricted cubic spline modeled treatment interval, with knots at 6, 32, and 87.4 days and a 32-day reference. All 98 patients, including the 857-day observation, were included in spline fitting; the plotted range was restricted to 0–180 days. Physical and EQD2 Vx metrics were fitted separately with continuous interval, with effects expressed per percentage point. Correlated dose metrics were not included simultaneously. Additional models and diagnostics were exploratory, without multiplicity adjustment. Supplementary logistic models comprised dose-only model (MLD_EQD2_ alone) and dose + interval model (MLD_EQD2_ plus interval per 10 days). These models used the 96 patients with RP or complete 90-day event-free follow-up. The dose + interval model included two slopes and an intercept.

Logistic internal validation used 50 repeats of stratified 10-fold cross-validation and 1,000 ordinary case bootstraps (20, 21, 24). Nested bootstrapping used 1,000 outer samples and 500 inner samples per outer sample. This yielded approximate percentile 95% intervals for optimism-corrected AUC, Brier score, calibration slope, joint calibration intercept, and calibration-in-the-large. These intervals are conditional on the fixed model specification and empirical sample. Uniform shrinkage was based on the bootstrap-corrected calibration slope, with the intercept re-estimated to preserve the overall event rate; the resulting equation was not independently validated. Decision-curve net benefit (25) was calculated from all 96 out-of-fold predictions within each repetition and averaged across 50 repetitions. Variation across fold assignments describes resampling variability rather than population-level confidence intervals. Model specifications, coefficients, shrinkage, and internal-validation performance are provided in **Supplementary Table 2**; calibration and decision curves appear in **Supplementary Figures S1, S2**.

### 2.7 Sensitivity and exploratory analyses

Sensitivity analyses evaluated physical MLD and individual Vx metrics, alternative covariate adjustment, interval restrictions, and dosimetric uncertainty. Dosimetric exclusions removed 18 patients identified during initial quality assurance (n = 80), with an additional analysis excluding one further course with fraction-count uncertainty (n = 79). Interval analyses excluded values >180 days (n = 94) or the longest observation (n = 97). a fractionation-restricted analysis refitted the primary model in patients treated with fraction sizes ≤3 Gy to reduce sensitivity to larger fraction sizes. Alternative upper fraction-size thresholds of 2.5, 4, 5, and 6 Gy were examined for consistency. An expanded model added preLYM and GTV-P, and a separate interval model adjusted for MLD and GTV-P. Cause-specific Cox regression and leave-one-out analyses assessed alternative hazard specifications and individual influence. The supplementary logistic analysis estimates odds among patients with an observed binary outcome and has a different estimand from the competing-risk analysis.

Dose and interval were modeled continuously in the primary analyses. Exploratory grouped analyses used a data-informed 30-day interval split and an MLD_EQD2_ split of 7.18 Gy. The latter was derived from the same cohort using ordinary binary ROC analysis using the Youden criterion. This derivation included all 98 patients, classifying 26 as RP and 72 as non-RP, including the two competing deaths. It did not account for event time or competing mortality. Grouped Aalen–Johansen curves and Gray tests subsequently accounted for competing mortality (**Supplementary Table 6**; **Supplementary Figure S3**). Both splits were exploratory and require independent validation before clinical interpretation.

### 2.8 Reporting

Reporting was guided by STROBE (26). TRIPOD-oriented reporting items were also considered for the supplementary logistic models and their internal validation. Corresponding manuscript locations and data limitations are summarized in a separate reporting checklist.

## 3 Results

### 3.1 Patient characteristics and event spectrum

Reconstructed physical and EQD2 MLD and V5–V30 were available for all 98 patients. Within 90 days, 26 patients developed grade ≥2 RP, 70 remained event-free, and two died before RP. No event-free observations were censored before day 90. The Aalen–Johansen RP cumulative incidence was 26.5% (pointwise normal-approximation 95% CI 17.7%–35.3%). Median recorded observation duration was 209.5 days (IQR 149–250.5; range 30–607), based on the latest valid clinical observation or exact death date. Recorded ICI cycle counts were available for 97 patients, with a median of 5 cycles (IQR, 3–12). Core characteristics are presented in **Table 1**, complete characteristics in **Supplementary Table 7**, and dose distributions in **Figure 2**. The supplementary logistic cohort comprised 96 patients.

**Table 1:** Patient characteristics by 90-day outcome in the primary cohort (n = 98).

Variable	Total (n=98)	Event-free (n=70)	RP ≥2 (n=26)	Death (n=2)
Male sex, n (%)	90 (91.8%)	64 (91.4%)	24 (92.3%)	2 (100.0%)
Age, mean ± SD	64.45 ± 9.39	64.36 ± 9.19	64.38 ± 10.33	68.50 ± 4.95
Baseline lung disease, n (%)	52 (53.1%)	39 (55.7%)	12 (46.2%)	1 (50.0%)
NSCLC, n (%)	83 (84.7%)	62 (88.6%)	20 (76.9%)	1 (50.0%)
SCLC, n (%)	13 (13.3%)	7 (10.0%)	5 (19.2%)	1 (50.0%)
Mixed carcinoma/other¹, n (%)	2 (2.0%)	1 (1.4%)	1 (3.8%)	0 (0.0%)
TNM stage I, n (%)	1 (1.0%)	1 (1.4%)	0 (0.0%)	0 (0.0%)
TNM stage III, n (%)	55 (56.1%)	41 (58.6%)	14 (53.8%)	0 (0.0%)
TNM stage IV, n (%)	42 (42.9%)	28 (40.0%)	12 (46.2%)	2 (100.0%)
ICI–RT interval (days), median [IQR]	32.00 [22.25, 48.50]	34.00 [25.25, 57.00]	26.00 [7.75, 37.75]	28.00 [23.50, 32.50]
Fraction dose (Gy), mean ± SD	3.01 ± 1.66	3.05 ± 1.81	2.90 ± 1.27	3.00 ± 0.00
Completed fraction count, mean ± SD	20.17 ± 8.84	20.94 ± 9.26	18.69 ± 7.58	12.50 ± 3.54
GTV-P (cm^3), mean ± SD	162.95 ± 129.56	140.11 ± 115.57	215.98 ± 148.90	272.78 ± 136.26
MLD_EQD2_ (Gy), mean ± SD	6.86 ± 2.31	6.41 ± 2.28	8.06 ± 2.00	6.87 ± 2.63
V5_EQD2_ (%), mean ± SD	28.32 ± 10.90	26.11 ± 10.99	34.35 ± 8.57	27.27 ± 7.68
V20_EQD2_ (%), mean ± SD	10.37 ± 4.41	9.62 ± 4.23	12.35 ± 4.47	11.24 ± 3.19
Physical MLD (Gy), mean ± SD	7.84 ± 2.82	7.46 ± 2.98	8.91 ± 2.13	7.34 ± 2.72
Physical V5 (%), mean ± SD	35.54 ± 13.47	33.14 ± 13.93	42.09 ± 10.14	34.18 ± 11.44
Physical V20 (%), mean ± SD	12.32 ± 5.69	11.59 ± 5.86	14.34 ± 4.93	11.68 ± 3.81

Values are counts (%) or mean ± SD unless median [IQR] is indicated. Event-free patients completed 90-day follow-up without RP; deaths before RP are shown separately. Baseline pulmonary disease is a composite variable with incomplete subtype information. Fraction counts represent completed treatment. ¹One mixed carcinoma occurred in the RP group and one other histology in the event-free group. Complete characteristics are provided in **Supplementary Table 7**.

**Figure 2:**
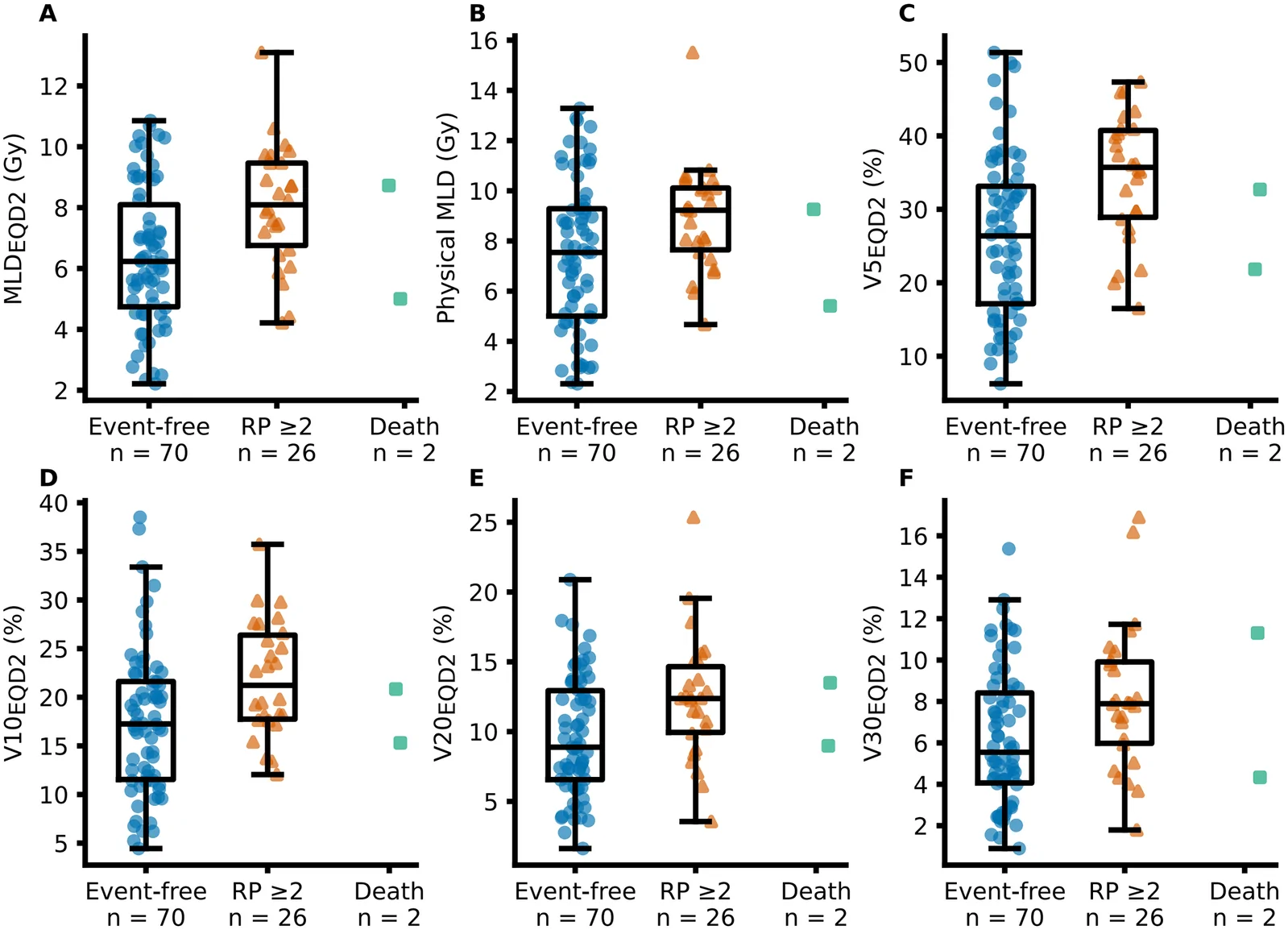
Reconstructed lung-dose distributions by 90-day outcome. **(A)** MLDEQD2 (Gy); **(B)** physical MLD (Gy); **(C)** V5EQD2 (%); **(D)** V10EQD2 (%); **(E)** V20EQD2 (%); and **(F)** V30EQD2 (%). Groups comprise event-free patients (n = 70), patients with grade ≥2 RP (n = 26), and deaths before RP (n = 2). Points represent individual patients. Boxes show medians and IQRs, with 1.5-IQR whiskers; the two competing deaths are displayed individually. Physical and EQD2 metrics use the same lung ROI and completed-course scaling.

### 3.2 Collinearity assessment and regression analysis

Spearman correlations among dosimetric indices, treatment interval, GTV-P, and preLYM are shown in **Figure 3**. The correlation between interval and MLD_EQD2_ was −0.002, and their variance inflation factors in the primary model were 1.002. Median interval was 32 days (IQR 22.25–48.50; range 0–857). The interval SHR per 10 days was 0.802 without adjustment (95% CI 0.673–0.957; P = 0.015). It was 0.784 after MLD adjustment (95% CI 0.639–0.963; P = 0.020) and 0.796 after additional GTV-P adjustment (95% CI 0.652–0.971; P = 0.025). MLD adjustment changed the absolute interval coefficient by 10.6% (**Supplementary Table 5**).

**Figure 3:**
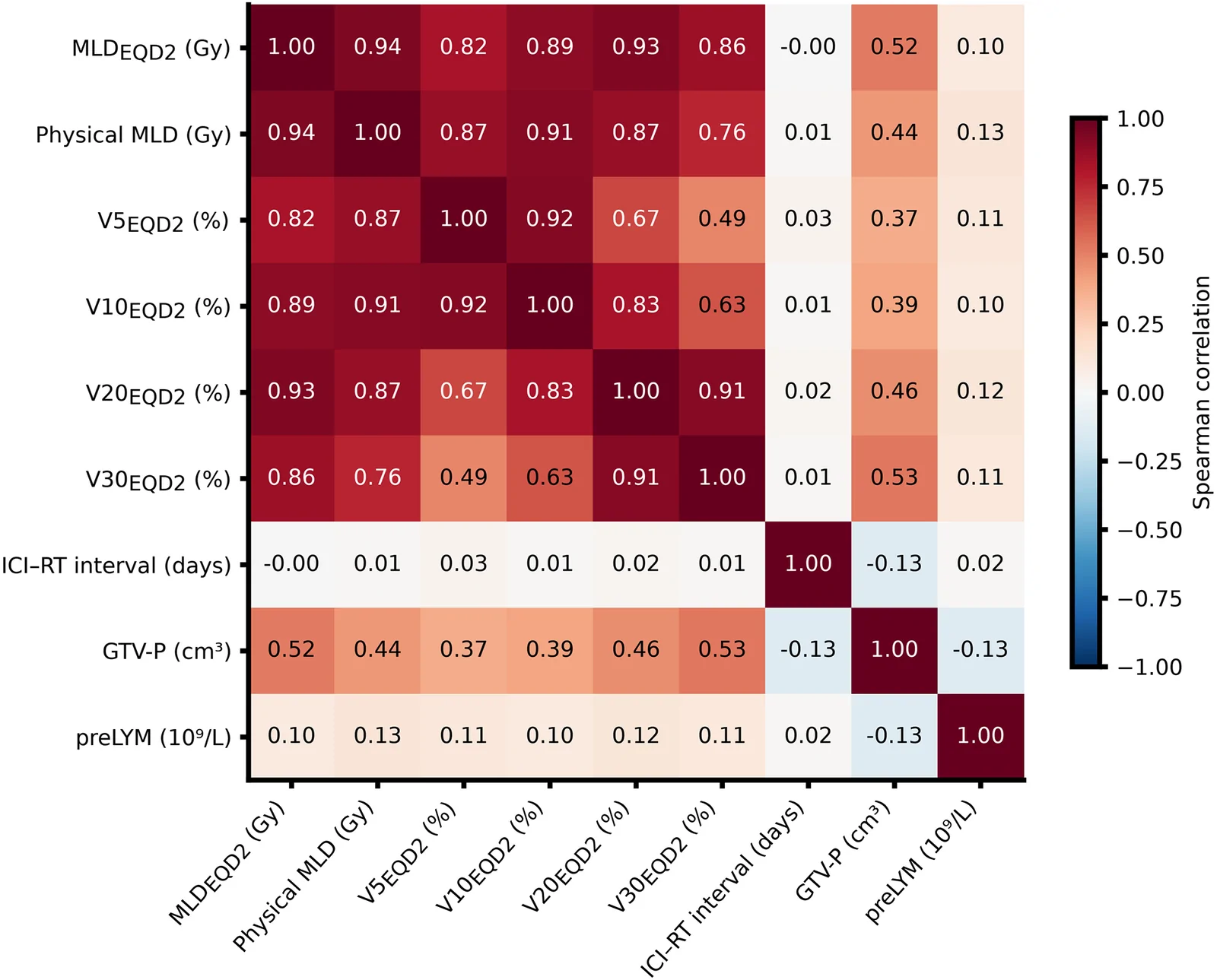
Spearman correlations among dosimetric and clinical variables in 98 patients. MLD_EQD2_ is the volume-weighted mean of voxel-wise EQD2. Vx_EQD2_ denotes the percentage of analysis lung volume with voxel EQD2 ≥x Gy.

The unadjusted SHR for MLD_EQD2_ was 1.344 per Gy (95% CI 1.117–1.618; P = 0.002). In the primary adjusted model, MLD_EQD2_ remained associated with RP (SHR 1.339 per Gy, 95% CI 1.113–1.612; P = 0.002). The corresponding interval SHR was 0.784 per 10 days (95% CI 0.639–0.963; P = 0.020). Complete coefficients are presented in **Table 2**, and separate interval-adjusted dosimetric models are summarized in **Table 3**.

**Table 2:** Coefficients of the primary Fine–Gray model in 98 patients, including 26 RP events and two competing deaths.

Term	Coefficient	SE	SHR (95% CI)	P
MLD_EQD2_ per Gy	0.292	0.095	1.339 (1.113–1.612)	0.002
Interval per 10 days	-0.243	0.105	0.784 (0.639–0.963)	0.020

MLD_EQD2_ is expressed per Gy and treatment interval per 10 days. SHR, subdistribution hazard ratio; SE, standard error.

**Table 3:** Separate interval-adjusted Fine–Gray models for lung-dose metrics in 98 patients.

Dose metric	Adjusted SHR (95% CI)	P
MLD_EQD2_ per Gy	1.339 (1.113–1.612)	0.002
Physical MLD per Gy	1.197 (1.036–1.382)	0.015
V5_EQD2_ per percentage point	1.065 (1.026–1.106)	<0.001
V10_EQD2_ per percentage point	1.071 (1.016–1.128)	0.010
V20_EQD2_ per percentage point	1.139 (1.028–1.260)	0.012
V30_EQD2_ per percentage point	1.151 (1.028–1.288)	0.015
V5 physical per percentage point	1.046 (1.017–1.076)	0.002
V10 physical per percentage point	1.059 (1.017–1.102)	0.005
V20 physical per percentage point	1.083 (1.003–1.170)	0.041
V30 physical per percentage point	1.089 (0.978–1.212)	0.121

MLD effects are expressed per Gy and Vx effects per percentage point. Each model includes one dose metric and continuous treatment interval. P values are exploratory and unadjusted for multiplicity.

**Figure 4A** shows the cumulative incidence of RP and competing death. **Figure 4B** presents fitted 90-day RP incidence across continuous MLD_EQD2_ values at the median treatment interval. Exploratory cumulative incidence curves stratified by dose and interval are provided in **Supplementary Figure 3**.

**Figure 4:**
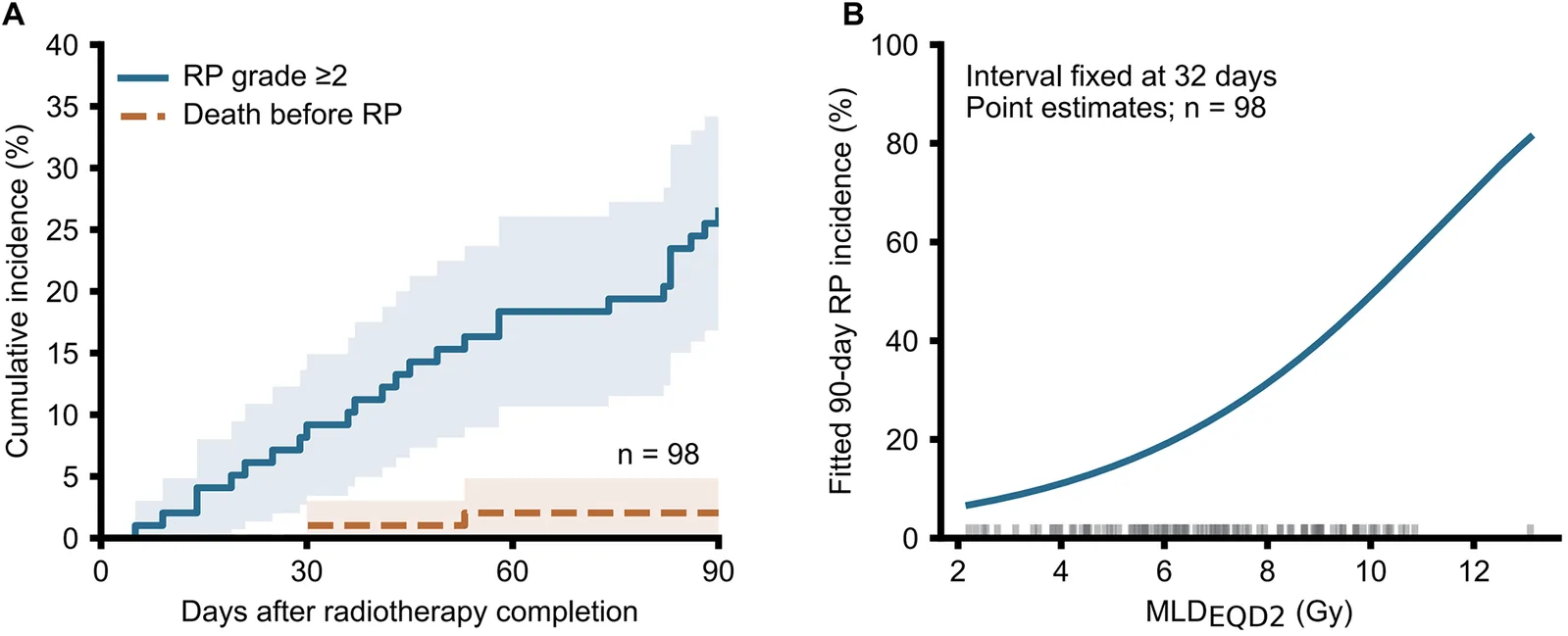
Cumulative incidence and continuous dose association. **(A)**, Aalen–Johansen cumulative incidence of grade ≥2 RP and death before RP in 98 patients. Shading indicates pointwise normal-approximation 95% intervals bounded to 0–100%. **(B)**, fitted 90-day RP incidence from the Fine–Gray model across MLD_EQD2_ values, with interval held at its median. Rug marks indicate observed doses. Panel B displays model point estimates without confidence intervals; predictive performance has not been validated.

### 3.3 Model diagnostics

Time-interaction tests yielded P = 0.875 for MLD, P = 0.202 for interval, and P = 0.441 jointly. The added quadratic MLD term had P = 0.650. Residual plots are shown in **Figure 5**; time-varying estimates appear in **Supplementary Table 4**. The adjusted interval spline yielded an overall P = 0.050 (2 df), with no evidence of departure from linearity (P = 0.934; 1 df), supporting use of the linear interval term in the primary model. Analyses excluding long intervals gave similar results with either fixed or recalculated knots (**Supplementary Table 5**; **Supplementary Figure S4**).

**Figure 5:**
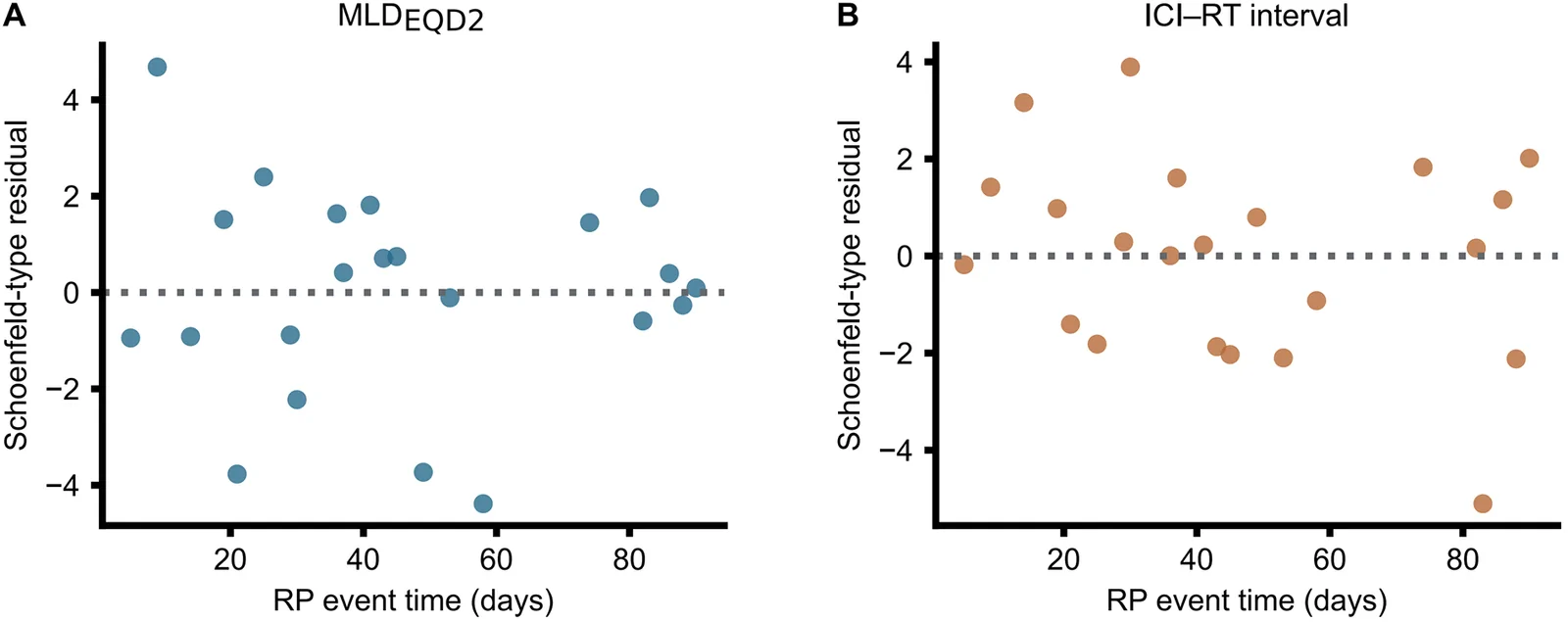
Schoenfeld-type residuals from the primary Fine–Gray model evaluated at RP event times, with tied events handled within the Fine–Gray model. **(A)**, MLD_EQD2_; **(B)**, interval per 10 days. The horizontal line denotes zero. Time-interaction P values were 0.875 for MLD and 0.202 for interval (joint P = 0.441).

For the supplementary dose + interval logistic model, bootstrap-corrected AUC was 0.760 (approximate 95% interval 0.654–0.868), and Brier score was 0.170 (0.119–0.207). The corrected calibration slope was 0.957 (0.920–1.009). Mean cross-validated AUC, Brier score, and calibration slope were 0.746, 0.168, and 0.798, respectively. At a 10% threshold, mean out-of-fold net benefit was 0.183 for dose + interval model and 0.190 for treating all patients. At 20%, the corresponding values were 0.143 and 0.089 (**Supplementary Table 2**; **Supplementary Figures S1, S2**).

### 3.4 Sensitivity analyses

Physical MLD was associated with RP after interval adjustment (SHR 1.197 per Gy, 95% CI 1.036–1.382; P = 0.015). The expanded model yielded an MLD_EQD2_ SHR of 1.250 per Gy (95% CI 1.039–1.505; P = 0.018). Dosimetric exclusions yielded SHRs of 1.315 in 80 patients (95% CI 1.072–1.614; P = 0.009) and 1.325 in 79 patients (95% CI 1.078–1.629; P = 0.007). Interval-restricted analyses showed consistent directions of association (**Supplementary Table 3**; **Figure 6**). Among 85 patients treated with fraction sizes ≤3 Gy (24 RP events, two competing deaths), the MLD_EQD2_ SHR was 1.282 per Gy (95% CI 1.048–1.567; P = 0.015) and the interval SHR 0.799 per 10 days (95% CI 0.645–0.989; P = 0.039). Physical MLD in this subgroup was 1.177 per Gy (95% CI 0.999–1.388; P = 0.052). Thresholds between 3 and 6 Gy gave similar estimates; restriction to ≤2.5 Gy left 44 patients with eight events and imprecise estimates (**Supplementary Table 3**). Physical V30 was not significantly associated with RP (P = 0.121). Cause-specific Cox estimates were 1.336 per Gy for MLD (95% CI 1.114–1.602) and 0.785 per 10 days for interval (95% CI 0.634–0.970). Leave-one-out SHRs ranged from 1.288 to 1.416 for MLD and 0.739 to 0.797 for interval, without direction reversal.

**Figure 6:**
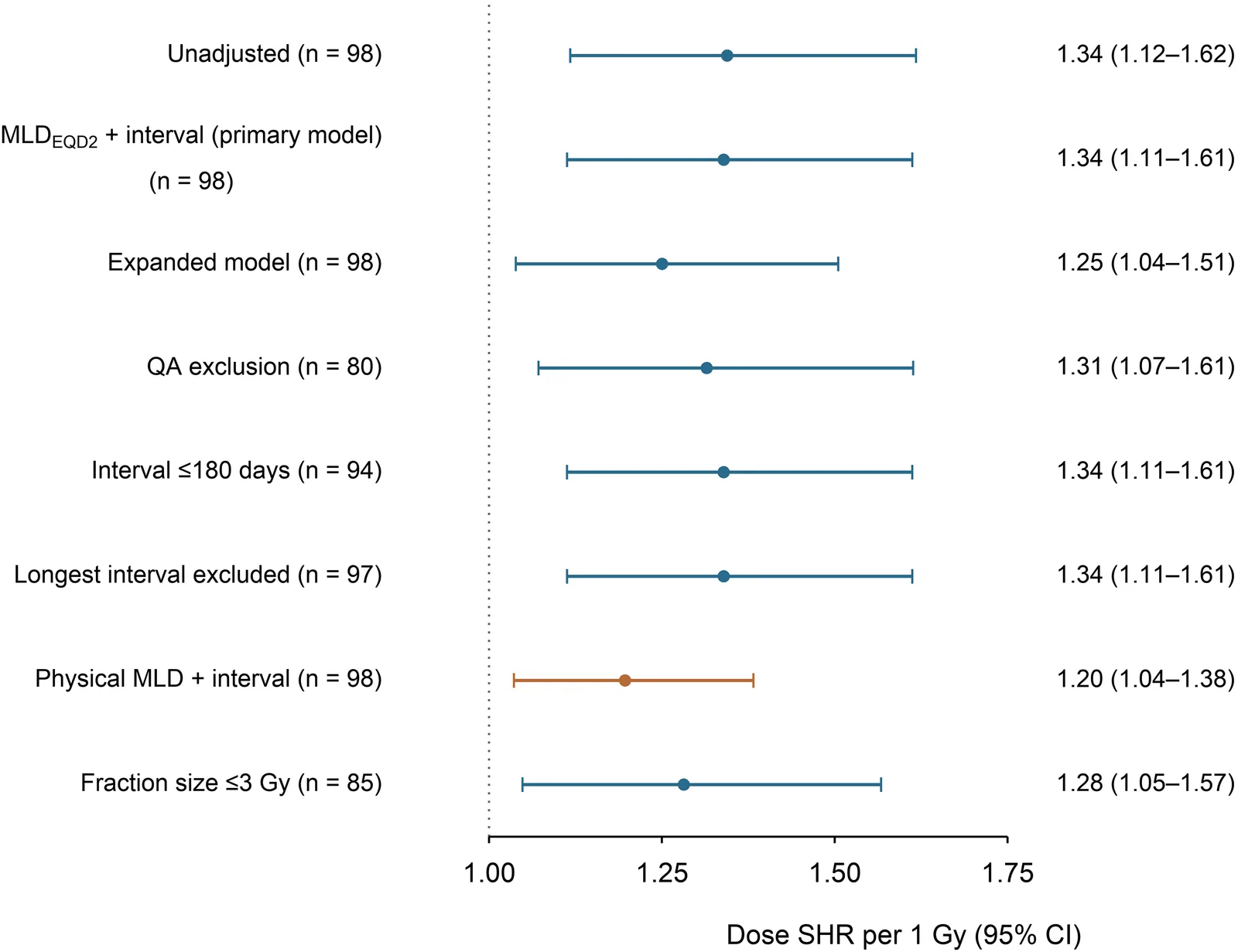
Dose associations in the primary and sensitivity Fine–Gray models. Points and bars indicate SHRs per Gy and Wald 95% confidence intervals. Blue indicates MLD_EQD2_ and orange indicates physical MLD. The expanded model additionally includes preLYM and GTV-P. Dosimetric exclusions leave 80 patients, including 21 RP events and one competing death. Interval restrictions leave 94 patients for intervals ≤180 days and 97 after excluding the longest interval. The final model restricts fraction size to ≤3 Gy (85 patients, 24 RP events, two competing deaths) and retains continuous MLD_EQD2_ and ICI–radiotherapy interval.

## 4 Discussion

In this retrospective cohort, the 90-day incidence of symptomatic RP after thoracic radiotherapy following ICI exposure was 26.5%. One grade 4 event and no grade 5 events were recorded. Higher MLD_EQD2_ was associated with RP after adjustment for continuous treatment interval. These findings quantify the dose–pneumonitis association in this specific treatment sequence. Comparisons with historical dose-volume studies, consolidation-immunotherapy cohorts, and concurrent regimens require caution because their populations, fractionation schedules, and ascertainment methods differ (8, 10, 15–17, 27–30).

The absence of an ICI-naive comparator precludes determining whether prior ICI exposure modifies the lung dose–response relationship. The association between shorter treatment intervals and RP may reflect treatment urgency, disease status, or incompletely captured systemic therapy exposure. Low collinearity between interval and MLD does not exclude these sources of residual confounding. The exploratory 30-day interval and 7.18-Gy dose splits therefore provide descriptive comparisons rather than clinical thresholds. These findings do not justify delaying otherwise indicated radiotherapy.

Voxel-wise EQD2 reconstruction incorporates variation in local dose per fraction across the lung. Because the transformation is nonlinear, converting an organ-level mean dose is not equivalent to averaging transformed voxel doses. Nevertheless, the assumed α/β of 3 Gy and repeated spatial dose distribution per fraction introduce uncertainty. The similar estimates in the ≤3 Gy/fraction subgroup suggest that the primary associations were not solely driven by the 13 higher-fraction-dose courses. This does not, however, demonstrate that the linear-quadratic conversion is valid across the full 1.5–10 Gy range, and the small number of events among patients receiving >3 Gy per fraction limits assessment of heterogeneity by fraction size. EQD2 also cannot account for differences in treatment intent or patient selection. The consistent physical-MLD association supports the overall direction of the dose relationship, but does not establish superiority of EQD2 over conventional dosimetry. The clinical role of reconstructed EQD2 metrics requires prospective evaluation alongside conventional physical-dose assessment.

The primary findings concern associations and do not establish a clinically validated risk calculator. Supplementary logistic validation yielded a bootstrap-corrected AUC of 0.760 and Brier score of 0.170. However, a mean cross-validated calibration slope of 0.798 suggests residual overfitting. These performance estimates apply to the 96-patient logistic model and cannot be transferred to the primary Fine–Gray model or the illustrative shrunken equation. Decision curves showed threshold-dependent net benefit, with no advantage over treating all patients at the 10% threshold. Clinical utility therefore remains uncertain. The limited power of diagnostic tests also warrants caution when interpreting the absence of significant time interactions.

This study has several limitations. The single-center retrospective design, 26 primary events, and clinical heterogeneity limit precision and generalizability. Exclusion of patients without post-radiotherapy clinical or imaging assessment may introduce selection bias. Incomplete documentation of clinical adjudication limits differentiation of radiation-associated, immune-mediated, infectious, and mixed pneumonitis (31). Knowledge of dose or field concordance during classification could inflate or otherwise distort the observed dose association. Mortality ascertainment was incomplete: one reported death lacked an exact date, and some death-status fields were missing. Estimates therefore depend on the accuracy of recorded follow-up. The interval distribution was highly skewed, with one observation at 857 days and sparse data at long intervals. Restricted analyses with fixed or recalculated spline knots gave similar findings, but estimates in the sparsely observed tail remain uncertain. Planning objectives and target margins could not be reliably recovered from the available records. Treatment intent, steroid exposure, pulmonary-disease subtypes, systemic-treatment timing, and subsequent ICI exposure could not be adequately incorporated into adjustment. The primary model was selected after initial analysis, and exploratory tests were not adjusted for multiplicity. Internal resampling does not account for all prior analytical selection, and external validation was unavailable.

CT-derived markers may complement clinical and dosimetric risk assessment. The CIPHER study evaluated pretreatment CT for predicting ICI-induced pneumonitis, which differs from the endpoint and treatment sequence examined here (32). Prospective studies could evaluate whether imaging-derived markers improve risk assessment when combined with clinical and dosimetric variables in patients receiving radiotherapy after ICI exposure.

## 5 Conclusion

Higher voxel-wise MLD_EQD2_ and shorter ICI–radiotherapy intervals were associated with grade ≥2 symptomatic pneumonitis in this retrospective cohort. These findings provide estimates specific to thoracic radiotherapy after ICI exposure, but cannot establish ICI-related radiosensitization without an ICI-naive comparator. Prospective studies with standardized ascertainment and independent validation are needed before these associations inform lung-dose constraints or treatment scheduling.

## Data Availability

The datasets presented in this article are not readily available because they contain patient clinical data and their sharing is restricted by institutional and ethics requirements. De-identified data and analysis code are available from the corresponding author upon reasonable request. Requests to access the datasets should be directed to Yuyin Le, yuyinle@126.com.
