# Supplementary material for: EQD2-adjusted mean lung dose and symptomatic pneumonitis after thoracic radiotherapy following immune checkpoint inhibitors in lung cancer: a single-center retrospective cohort study
Source: Front Oncol. 2026 Oct 9;16:1923622. doi: 10.3389/fonc.2026.1923622 (PMC13690549; doi:10.3389/fonc.2026.1923622)
Supplement: Supplementary file 1 [file DataSheet1.docx]

**Supplementary material**

The primary competing-risk cohort comprised 98 patients: 26 with RP, 70 with complete 90-day event-free follow-up, and two who died before RP. Supplementary logistic analyses included the 96 patients with an observed binary outcome. All supplementary analyses were exploratory.

## **Supplementary Methods**

Archived RTDOSE, RTPLAN and RTSTRUCT data were used for retrospective dose reconstruction. The lung contour definition was verified in all 98 patients: every combined-lung region of interest represented bilateral lungs excluding the GTV or ITV, rather than the PTV. These regions were used directly without further target subtraction. Contours were rasterized on the CT grid and transferred to the native dose grid using nearest-neighbor mask resampling. Isotropic dose-grid spacing was 3 mm in 88 patients, 2 mm in six, and 4 mm in four. Dose-grid voxel volumes provided the weights for dose-volume calculations.

For incomplete courses, the planned dose distribution was multiplied by the ratio of completed to planned fractions, assuming an unchanged spatial dose distribution across fractions. Total physical dose at each lung voxel was converted to EQD2 using the completed fraction count and an α/β ratio of 3 Gy, as defined in Section 2.3 of the main manuscript. Mean lung EQD2 was calculated after voxel-wise conversion by weighting each voxel by its volume. EQD2-based Vx was the percentage of lung volume at or above the specified EQD2 threshold. Physical MLD and Vx were calculated from the same lung region before conversion. This reconstruction estimates completed-course dose under the stated assumptions; it does not measure dose delivered in each fraction.

Dose reconstruction used Python 3.11.13. Competing-risk analyses used R 4.2.2 with cmprsk 2.2-12.

## **Supplementary Table S1. Logistic dose associations in 96 patients**

Each dose metric was fitted separately, either unadjusted or adjusted for continuous interval per 10 days. The MLD_EQD2_ specifications correspond to the dose-only and dose + interval logistic models used for validation. MLD effects are expressed per Gy and Vx effects per percentage point. P values are unadjusted for multiplicity. The two deaths before RP were excluded from these supplementary logistic analyses.

| **Dose metric** | **Model** | **OR (95% CI)** | **P** |
| --- | --- | --- | --- |
| MLD_EQD2_ | Unadjusted | 1.403 (1.121–1.756) | 0.003 |
| MLD_EQD2_ | Interval-adjusted | 1.422 (1.126–1.796) | 0.003 |
| Physical MLD | Unadjusted | 1.212 (1.019–1.442) | 0.030 |
| Physical MLD | Interval-adjusted | 1.236 (1.024–1.492) | 0.027 |
| V5_EQD2_ (%) | Unadjusted | 1.080 (1.029–1.133) | 0.002 |
| V5_EQD2_ (%) | Interval-adjusted | 1.092 (1.035–1.152) | 0.001 |
| V10_EQD2_ (%) | Unadjusted | 1.091 (1.020–1.166) | 0.011 |
| V10_EQD2_ (%) | Interval-adjusted | 1.108 (1.028–1.194) | 0.007 |
| V20_EQD2_ (%) | Unadjusted | 1.155 (1.034–1.291) | 0.011 |
| V20_EQD2_ (%) | Interval-adjusted | 1.161 (1.035–1.303) | 0.011 |
| V30_EQD2_ (%) | Unadjusted | 1.177 (1.026–1.349) | 0.020 |
| V30_EQD2_ (%) | Interval-adjusted | 1.174 (1.020–1.352) | 0.025 |
| V5 physical (%) | Unadjusted | 1.056 (1.016–1.097) | 0.006 |
| V5 physical (%) | Interval-adjusted | 1.063 (1.019–1.108) | 0.004 |
| V10 physical (%) | Unadjusted | 1.073 (1.018–1.131) | 0.008 |
| V10 physical (%) | Interval-adjusted | 1.084 (1.022–1.148) | 0.007 |
| V20 physical (%) | Unadjusted | 1.091 (1.003–1.186) | 0.041 |
| V20 physical (%) | Interval-adjusted | 1.098 (1.003–1.202) | 0.044 |
| V30 physical (%) | Unadjusted | 1.090 (0.978–1.214) | 0.118 |
| V30 physical (%) | Interval-adjusted | 1.087 (0.970–1.217) | 0.150 |

## **Supplementary Table S2. Logistic models, internal validation and shrinkage**

**A. Internal-validation performance of dose + interval model (n = 96; 26 events)**

Dose + interval model includes MLD_EQD2_ and continuous interval per 10 days. Performance is reported for the development sample, after optimism correction using 1,000 bootstraps, and across 50 repeats of stratified 10-fold cross-validation. Approximate 95% intervals for corrected estimates used 1,000 outer bootstrap samples with 500 inner samples each. These intervals are conditional on the fixed model and empirical sample and exclude uncertainty from earlier model selection and external transportability.

| **Metric** | **Apparent** | **Corrected (approx. 95% interval)** | **CV mean** |
| --- | --- | --- | --- |
| AUC | 0.774 | 0.760 (0.654–0.868) | 0.746 |
| Brier score | 0.159 | 0.170 (0.119–0.207) | 0.168 |
| Calibration slope | 1.000 | 0.957 (0.920–1.009) | 0.798 |
| Joint calibration intercept | 0.000 | -0.026 (-0.052–0.038) | -0.158 |
| Calibration-in-the-large | 0.000 | -0.002 (-0.019–0.039) | -0.006 |

Calibration slope and intercept were estimated jointly; calibration-in-the-large was estimated with the slope fixed at one. Performance estimates apply to the original dose + interval model, not the illustrative shrunken equation. Bootstrap optimism was the calibration slope in each bootstrap development sample minus the slope obtained by applying that fitted model to the original sample; mean optimism was subtracted from the apparent slope. The corrected slope was 0.957 (mean optimism 0.043), whereas the mean slope from each repetition’s pooled held-out predictions was 0.798. These estimates use different training samples and evaluation schemes and are not interchangeable; the lower cross-validated slope suggests residual overfitting.

## **B. Complete fixed logistic coefficients**

| **Model / term** | **β** | **SE** | **OR (95% CI)** | **P** |
| --- | --- | --- | --- | --- |
| dose-only model / Intercept | -3.4413 | 0.900409 | 0.032 (0.005–0.187) | <0.001 |
| dose-only model / MLD_EQD2_ per Gy | 0.3386 | 0.114518 | 1.403 (1.121–1.756) | 0.003 |
| dose + interval model / Intercept | -2.4735 | 0.959223 | 0.084 (0.013–0.552) | 0.010 |
| dose + interval model / MLD_EQD2_ per Gy | 0.3520 | 0.119009 | 1.422 (1.126–1.796) | 0.003 |
| dose + interval model / Interval per 10 days | -0.3178 | 0.143083 | 0.728 (0.550–0.963) | 0.026 |

The dose-only model includes MLD_EQD2_ alone, and the dose + interval model additionally includes continuous treatment interval. Coefficient intervals are Wald intervals. For intercepts, exp(β) denotes odds at the reference values of the predictors.

The unshrunk dose + interval model is logit(p) = −2.473579 + 0.352043 × MLD_EQD2_ − 0.317853 × interval_10_, where interval10 is the calendar-day interval divided by 10. The predicted probability is the inverse logit of this expression.

**C. Uniform shrinkage demonstration**

| **Shrunken dose + interval model term** | **β** | **exp(β) (approx. 95% interval)** | **P** |
| --- | --- | --- | --- |
| Intercept | -2.398670 | 0.091 (0.012–0.444) | Not applicable |
| MLD_EQD2_ per Gy | 0.336865 | 1.401 (1.160–1.869) | Not applicable |
| Interval per 10 days | -0.304149 | 0.738 (0.503–0.898) | Not applicable |

The uniform shrinkage factor was 0.9569. The intercept was re-estimated so that the mean predicted probability equaled 26/96. The shrunken equation is logit(p) = −2.398670 + 0.336865 × MLD_EQD2_ − 0.304149 × interval_10_. Coefficient intervals are outer-bootstrap percentiles, with shrinkage re-estimated within each outer sample. P values are not applicable to these estimates. The shrunken equation illustrates the shrinkage procedure and has not undergone independent performance validation.

## **Supplementary Table S3. Competing-risk and cause-specific robustness**

Models include continuous interval and MLD_EQD2_ unless physical MLD is specified. The expanded model additionally includes preLYM and GTV-P. Dosimetric sensitivity analyses exclude 18 patients identified during initial quality assurance (n = 80). The extended exclusion set additionally removes one course with initial fraction-count uncertainty (n = 79). SHRs describe subdistribution hazards, whereas cause-specific HRs describe hazards among patients remaining event-free.

| **Specification (n/RP/death)** | **Term** | **SHR (95% CI)** | **P** |
| --- | --- | --- | --- |
| Unadjusted (98/26/2) | MLD_EQD2_ per Gy | 1.344 (1.117–1.618) | 0.002 |
| Dosimetric exclusion (80/21/1) | MLD_EQD2_ per Gy | 1.315 (1.072–1.614) | 0.009 |
| Dosimetric exclusion (80/21/1) | Interval per 10 days | 0.795 (0.647–0.976) | 0.028 |
| Interval ≤180 days (94/26/2) | MLD_EQD2_ per Gy | 1.339 (1.113–1.612) | 0.002 |
| Interval ≤180 days (94/26/2) | Interval per 10 days | 0.784 (0.637–0.965) | 0.022 |
| Exclude longest (97/26/2) | MLD_EQD2_ per Gy | 1.339 (1.113–1.612) | 0.002 |
| Exclude longest (97/26/2) | Interval per 10 days | 0.784 (0.639–0.963) | 0.020 |
| Extended exclusion (79/21/1) | MLD_EQD2_ per Gy | 1.325 (1.078–1.629) | 0.007 |
| Extended exclusion (79/21/1) | Interval per 10 days | 0.806 (0.659–0.986) | 0.036 |
| Extended exclusion (79/21/1) | Physical MLD per Gy | 1.187 (1.018–1.384) | 0.028 |
| Extended exclusion (79/21/1) | Interval per 10 days | 0.806 (0.662–0.981) | 0.032 |
| Dosimetric exclusion (80/21/1) | Physical MLD per Gy | 1.184 (1.015–1.382) | 0.031 |
| Dosimetric exclusion (80/21/1) | Interval per 10 days | 0.798 (0.653–0.975) | 0.027 |
| Expanded (98/26/2) | MLD_EQD2_ per Gy | 1.250 (1.039–1.505) | 0.018 |
| Expanded (98/26/2) | Interval per 10 days | 0.787 (0.637–0.974) | 0.028 |
| Expanded (98/26/2) | preLYM per 10⁹/L | 1.742 (1.137–2.668) | 0.011 |
| Expanded (98/26/2) | GTV-P per cm³ | 1.002 (1.000–1.005) | 0.074 |
| Fraction size ≤3 Gy (85/24/2) | MLDEQD2 per Gy | 1.282 (1.048–1.567) | 0.015 |
| Fraction size ≤3 Gy (85/24/2) | Interval per 10 days | 0.799 (0.645–0.989) | 0.039 |
| Fraction size ≤3 Gy, physical-dose model (85/24/2) | Physical MLD per Gy | 1.177 (0.999–1.388) | 0.052 |
| Fraction size ≤3 Gy, physical-dose model (85/24/2) | Interval per 10 days | 0.798 (0.641–0.992) | 0.042 |

**Cause-specific and fraction-size analyses (Supplementary Table S3 continued)**

| **Cause-specific Cox (n = 98)** | **β (SE)** | **HR (95% CI)** | **P** |
| --- | --- | --- | --- |
| MLD_EQD2_ per Gy | 0.290 (0.093) | 1.336 (1.114–1.602) | 0.002 |
| Interval per 10 days | -0.242 (0.108) | 0.785 (0.634–0.970) | 0.025 |

| **Leave-one-out term** | **Fits** | **SHR range** | **Direction reversals** |
| --- | --- | --- | --- |
| Interval per 10 days | 98 | 0.739–0.797 | 0 |
| MLD_EQD2_ per Gy | 98 | 1.288–1.416 | 0 |

The largest standardized coefficient changes were 0.587 for MLD and 0.568 for interval, both after removing the same observation. Coefficients and the primary baseline cumulative subdistribution hazard are available under the manuscript Data Availability Statement.

**Fraction-size threshold sensitivity (Supplementary Table S3 continued)**

| **Upper fraction size (Gy)** | **n / RP / death** | **MLD_EQD2_ SHR (95% CI)** | **P** | **Interval SHR (95% CI)** | **P** |
| --- | --- | --- | --- | --- | --- |
| 2.5 | 44/8/0 | 1.121 (0.727–1.729) | 0.604 | 0.761 (0.525–1.105) | 0.151 |
| 3 | 85/24/2 | 1.282 (1.048–1.567) | 0.015 | 0.799 (0.645–0.989) | 0.039 |
| 4 | 86/24/2 | 1.282 (1.048–1.567) | 0.015 | 0.799 (0.646–0.988) | 0.039 |
| 5 | 90/25/2 | 1.294 (1.067–1.570) | 0.009 | 0.793 (0.643–0.978) | 0.030 |
| 6 | 91/25/2 | 1.303 (1.076–1.577) | 0.007 | 0.794 (0.643–0.981) | 0.032 |

Each model includes MLD_EQD2_ per Gy and interval per 10 days. All models converged. Only eight RP events remained at ≤2.5 Gy, limiting precision. All threshold analyses are exploratory and unadjusted for multiplicity.

## **Supplementary Table S4. Fine–Gray diagnostics and time-varying sensitivity estimates**

| **Diagnostic** | **Wald χ²** | **df** | **P** |
| --- | --- | --- | --- |
| Joint covariate × t/90 | 1.639 | 2 | 0.441 |
| MLD × t/90 | 0.025 | 1 | 0.875 |
| Interval × t/90 | 1.630 | 1 | 0.202 |
| Added centered MLD² | 0.205 | 1 | 0.650 |

The time-varying model includes MLD, interval, and both predictor-by-time interactions, giving four slopes. The quadratic term evaluates dose nonlinearity. These exploratory diagnostics have limited power in a cohort with 26 events.

| **Event day** | **Term** | **SHR (pointwise 95% CI)** |
| --- | --- | --- |
| 0 | MLD_EQD2_ per Gy | 1.376 (0.936–2.022) |
| 0 | Interval per 10 days | 0.958 (0.710–1.294) |
| 30 | MLD_EQD2_ per Gy | 1.358 (1.059–1.741) |
| 30 | Interval per 10 days | 0.839 (0.699–1.007) |
| 60 | MLD_EQD2_ per Gy | 1.340 (1.122–1.599) |
| 60 | Interval per 10 days | 0.735 (0.576–0.938) |
| 90 | MLD_EQD2_ per Gy | 1.322 (1.043–1.676) |
| 90 | Interval per 10 days | 0.643 (0.426–0.970) |

SHRs are evaluated at the indicated times after radiotherapy completion; day 0 represents a boundary estimate. Corresponding residuals appear in main Figure 5.

## **Supplementary Table S5. Continuous-interval analysis, collinearity and adjusted spline**

Continuous interval analyses include all 98 patients, with a median interval of 32 days (IQR 22.25–48.50; range 0–857). Linear and spline models use the same population and MLD adjustment. The additional GTV-P model examines sensitivity to tumor-volume adjustment.

| **Interval specification** | **Term** | **SHR (95% CI)** | **P** |
| --- | --- | --- | --- |
| Unadjusted | Interval per 10 days | 0.802 (0.673–0.957) | 0.015 |
| MLD-adjusted | MLD_EQD2_ per Gy | 1.339 (1.113–1.612) | 0.002 |
| MLD-adjusted | Interval per 10 days | 0.784 (0.639–0.963) | 0.020 |
| MLD- and GTV-P-adjusted | MLD_EQD2_ per Gy | 1.295 (1.086–1.544) | 0.004 |
| MLD- and GTV-P-adjusted | Interval per 10 days | 0.796 (0.652–0.971) | 0.025 |
| MLD- and GTV-P-adjusted | GTV-P per 50 cm³ | 1.096 (0.981–1.225) | 0.106 |
| MLD-adjusted 30-day group | MLD_EQD2_ per Gy | 1.377 (1.139–1.665) | <0.001 |
| MLD-adjusted 30-day group | Interval ≥30 vs <30 days | 0.346 (0.151–0.791) | 0.012 |

| **Adjustment** | **Interval β** | **Absolute β change (%)** | **Interval VIF** |
| --- | --- | --- | --- |
| Unadjusted | -0.220 | 0.00 | 1.000 |
| Adjusted for MLD_EQD2_ | -0.243 | 10.59 | 1.002 |
| Adjusted for MLD_EQD2_ and GTV-P | -0.228 | 3.77 | 1.011 |

The Spearman correlation between interval and MLD_EQD2_ was −0.002 (bootstrap 95% interval −0.205 to 0.198). Low variance inflation factors indicate limited collinearity between the included predictors but do not exclude unmeasured confounding.

| **Spline population** | **n** | **Overall P (2 df)** | **Nonlinearity P (1 df)** |
| --- | --- | --- | --- |
| All 98 patients | 98 | 0.050 | 0.934 |
| Exclude intervals >180 days | 94 | 0.056 | 0.922 |
| Exclude 857-day observation | 97 | 0.050 | 0.934 |
| Exclude 857-day observation; recalculated knots | 97 | 0.048 | 0.887 |
| Exclude intervals >180 days; recalculated knots | 94 | 0.052 | 0.843 |

The first three rows use fixed full-cohort knots at 6, 32, and 87.4 days. The excluded subsets contain 97 and 94 patients, respectively, and were refitted separately. The nearly identical P values after excluding the 857-day observation reflect its negligible fitted risk contribution. Recalculating the subset knots gives 6, 32, and 75.4 days for n = 97 and 6, 31, and 67.5 days for n = 94, with no evidence of nonlinearity in either analysis. The full-cohort spline uses 32 days as the reference and is displayed over 0–180 days in Figure S4. All tests are exploratory and unadjusted for multiplicity; these results do not identify a discrete interval threshold.

## **Supplementary Table S6. Exploratory grouped Aalen–Johansen RP cumulative incidence**

| **Group** | **n / RP / death** | **90-day CIF % (pointwise 95% CI)** |
| --- | --- | --- |
| MLD_EQD2_ <7.18 Gy | 57/7/1 | 12.28 (3.68–20.88) |
| MLD_EQD2_ ≥7.18 Gy | 41/19/1 | 46.34 (30.81–61.87) |
| Interval <30 days | 45/17/1 | 37.78 (23.40–52.15) |
| Interval ≥30 days | 53/9/1 | 16.98 (6.77–27.20) |

Gray tests yielded P < 0.001 for dose groups and P = 0.023 for interval groups, without multiplicity adjustment. The 30-day split was data-informed. The 7.18-Gy split was derived from ordinary binary ROC analysis using the same 98 patients and a Youden midpoint of 7.18 Gy. That derivation classified 26 patients as RP and 72 as non-RP, including two competing deaths, without accounting for event time or competing mortality. The subsequent Aalen–Johansen analysis accounts for competing mortality, but does not provide independent validation of either cutoff.

Supplementary Table S7. Patient characteristics by 90-day outcome in the primary cohort (n = 98). Values are counts (%) or mean ± SD unless median [IQR] is indicated. Event-free patients completed 90-day follow-up without RP; deaths before RP are shown separately. Baseline pulmonary disease is a composite variable with incomplete subtype information. ICI class is based on recorded categories; specific agents were unavailable. Fraction counts represent completed treatment. ¹One mixed carcinoma occurred in the RP group and one other histology in the event-free group.

| **Variable** | **Total (n=98)** | **Event-free (n=70)** | **RP ≥2 (n=26)** | **Death (n=2)** |
| --- | --- | --- | --- | --- |
| Sex, n (%) |  |  |  |  |
| Male | 90 (91.8%) | 64 (91.4%) | 24 (92.3%) | 2 (100.0%) |
| Female | 8 (8.2%) | 6 (8.6%) | 2 (7.7%) | 0 (0.0%) |
| Age, mean ± SD | 64.45 ± 9.39 | 64.36 ± 9.19 | 64.38 ± 10.33 | 68.50 ± 4.95 |
| Smoking status, n (%) |  |  |  |  |
| Never | 29 (29.6%) | 22 (31.4%) | 7 (26.9%) | 0 (0.0%) |
| Former | 42 (42.9%) | 29 (41.4%) | 12 (46.2%) | 1 (50.0%) |
| Current | 27 (27.6%) | 19 (27.1%) | 7 (26.9%) | 1 (50.0%) |
| Baseline lung disease, n (%) |  |  |  |  |
| No | 46 (46.9%) | 31 (44.3%) | 14 (53.8%) | 1 (50.0%) |
| Yes | 52 (53.1%) | 39 (55.7%) | 12 (46.2%) | 1 (50.0%) |
| ECOG status, n (%) |  |  |  |  |
| 0 | 3 (3.1%) | 2 (2.9%) | 1 (3.8%) | 0 (0.0%) |
| 1 | 86 (87.8%) | 61 (87.1%) | 24 (92.3%) | 1 (50.0%) |
| 2 | 9 (9.2%) | 7 (10.0%) | 1 (3.8%) | 1 (50.0%) |
| Histology subtype, n (%) |  |  |  |  |
| NSCLC | 83 (84.7%) | 62 (88.6%) | 20 (76.9%) | 1 (50.0%) |
| SCLC | 13 (13.3%) | 7 (10.0%) | 5 (19.2%) | 1 (50.0%) |
| Mixed carcinoma / other¹ | 2 (2.0%) | 1 (1.4%) | 1 (3.8%) | 0 (0.0%) |
| TNM stage, n (%) |  |  |  |  |
| I | 1 (1.0%) | 1 (1.4%) | 0 (0.0%) | 0 (0.0%) |
| III | 55 (56.1%) | 41 (58.6%) | 14 (53.8%) | 0 (0.0%) |
| IV | 42 (42.9%) | 28 (40.0%) | 12 (46.2%) | 2 (100.0%) |
| ICI type, n (%) |  |  |  |  |
| PD-1 | 85 (86.7%) | 61 (87.1%) | 22 (84.6%) | 2 (100.0%) |
| PD-L1 | 13 (13.3%) | 9 (12.9%) | 4 (15.4%) | 0 (0.0%) |
| ICI–RT interval (days), median [IQR] | 32.00 [22.25, 48.50] | 34.00 [25.25, 57.00] | 26.00 [7.75, 37.75] | 28.00 [23.50, 32.50] |
| Prior gemcitabine, n (%) |  |  |  |  |
| No | 80 (81.6%) | 55 (78.6%) | 23 (88.5%) | 2 (100.0%) |
| Yes | 18 (18.4%) | 15 (21.4%) | 3 (11.5%) | 0 (0.0%) |
| Lower-lobe tumor, n (%) |  |  |  |  |
| No | 68 (69.4%) | 50 (71.4%) | 16 (61.5%) | 2 (100.0%) |
| Yes | 30 (30.6%) | 20 (28.6%) | 10 (38.5%) | 0 (0.0%) |
| Mediastinal LN irradiation, n (%) |  |  |  |  |
| No | 46 (46.9%) | 35 (50.0%) | 10 (38.5%) | 1 (50.0%) |
| Yes | 52 (53.1%) | 35 (50.0%) | 16 (61.5%) | 1 (50.0%) |
| Fraction dose (Gy), mean ± SD | 3.01 ± 1.66 | 3.05 ± 1.81 | 2.90 ± 1.27 | 3.00 ± 0.00 |
| Completed fraction count, mean ± SD | 20.17 ± 8.84 | 20.94 ± 9.26 | 18.69 ± 7.58 | 12.50 ± 3.54 |
| GTV-P (cm^3), mean ± SD | 162.95 ± 129.56 | 140.11 ± 115.57 | 215.98 ± 148.90 | 272.78 ± 136.26 |
| MLD_EQD2_ (Gy), mean ± SD | 6.86 ± 2.31 | 6.41 ± 2.28 | 8.06 ± 2.00 | 6.87 ± 2.63 |
| V5_EQD2_ (%), mean ± SD | 28.32 ± 10.90 | 26.11 ± 10.99 | 34.35 ± 8.57 | 27.27 ± 7.68 |
| V10_EQD2_ (%), mean ± SD | 18.67 ± 7.23 | 17.49 ± 7.43 | 21.91 ± 5.92 | 18.07 ± 3.91 |
| V20_EQD2_ (%), mean ± SD | 10.37 ± 4.41 | 9.62 ± 4.23 | 12.35 ± 4.47 | 11.24 ± 3.19 |
| V30_EQD2_ (%), mean ± SD | 6.76 ± 3.43 | 6.22 ± 3.27 | 8.12 ± 3.52 | 7.82 ± 4.93 |
| preLYM (×10^9/L), median (IQR) | 1.50 [1.11, 1.87] | 1.35 [1.09, 1.81] | 1.65 [1.42, 2.05] | 0.89 [0.79, 0.98] |
| preNEU (×10^9/L), median (IQR) | 4.00 [2.90, 5.56] | 3.90 [2.94, 5.49] | 4.03 [2.62, 5.31] | 9.17 [7.53, 10.82] |
| preMONO (×10^9/L), median (IQR) | 0.55 [0.34, 0.70] | 0.53 [0.34, 0.69] | 0.54 [0.34, 0.74] | 0.65 [0.63, 0.66] |
| preALB (g/L), median (IQR) | 40.80 [38.10, 43.05] | 41.02 [38.16, 43.05] | 40.50 [38.10, 42.98] | 38.15 [36.97, 39.33] |
| NLR, median (IQR) | 2.44 [1.69, 4.21] | 2.42 [1.78, 4.45] | 2.43 [1.53, 3.13] | 11.65 [8.57, 14.72] |
| NPAR, median (IQR) | 0.10 [0.07, 0.15] | 0.10 [0.07, 0.14] | 0.10 [0.06, 0.15] | 0.25 [0.20, 0.30] |
| LMR, median (IQR) | 2.71 [2.18, 5.01] | 2.67 [2.15, 4.80] | 3.36 [2.34, 5.19] | 1.36 [1.25, 1.48] |
| Physical MLD (Gy), mean ± SD | 7.84 ± 2.82 | 7.46 ± 2.98 | 8.91 ± 2.13 | 7.34 ± 2.72 |
| Physical V5 (%), mean ± SD | 35.54 ± 13.47 | 33.14 ± 13.93 | 42.09 ± 10.14 | 34.18 ± 11.44 |
| Physical V20 (%), mean ± SD | 12.32 ± 5.69 | 11.59 ± 5.86 | 14.34 ± 4.93 | 11.68 ± 3.81 |
| Motion management, n (%) |  |  |  |  |
| None recorded | 73 (74.5%) | 47 (67.1%) | 24 (92.3%) | 2 (100.0%) |
| 4D-CT | 17 (17.3%) | 17 (24.3%) | 0 (0.0%) | 0 (0.0%) |
| Respiratory gating | 8 (8.2%) | 6 (8.6%) | 2 (7.7%) | 0 (0.0%) |

## **Supplementary Figure S1. Fixed-model out-of-fold calibration (dose-only and dose + interval models)**

The dose-only model includes MLD_EQD2_ alone, and the dose + interval model additionally includes continuous interval. Five approximately equal-sized groups of 19–20 patients were formed from each repetition’s held-out predictions. Points show group means across 50 repetitions, with lines connecting these descriptive summaries. Probabilities were not recalibrated before decision-curve analysis.

## **Supplementary Figure S2. Exploratory out-of-fold decision curves**

Net benefit was calculated from each complete set of 96 out-of-fold predictions and averaged across 50 repetitions. Shading indicates the 2.5th–97.5th percentiles across fold assignments, rather than population-level confidence intervals. The treat-all curve extends below the displayed axis at high thresholds. At 10%, the dose + interval model yielded lower net benefit than treating all patients (0.183 versus 0.190). Thresholds are illustrative; clinical utility requires independent evaluation.

## **Supplementary Figure S3. Exploratory grouped cumulative incidence**

A, MLD_EQD2_ <7.18 versus ≥7.18 Gy. B, interval <30 versus ≥30 days. Lines show Aalen–Johansen RP cumulative incidence; shading indicates pointwise normal-approximation 95% intervals bounded to 0–100%. Gray tests compare cumulative incidence between groups. Numbers at risk represent patients free of either event immediately before each indicated day. Both splits are exploratory (Table S6).

## **Supplementary Figure S4. MLD-adjusted restricted cubic spline for interval**

The three-knot spline is adjusted for MLD_EQD2_ in all 98 patients, including the 857-day observation. Knots are at 6, 32, and 87.4 days, with 32 days as the reference. The displayed range is 0–180 days. Shading indicates pointwise delta-method 95% intervals for relative SHRs; rug marks show observations within the displayed range. Overall P = 0.050 (2 df); nonlinearity P = 0.934 (1 df).
